# Comparative Mitogenomic Analysis of Two Longhorn Beetles (Coleoptera: Cerambycidae: Lamiinae) with Preliminary Investigation into Phylogenetic Relationships of Tribes of Lamiinae

**DOI:** 10.3390/insects12090820

**Published:** 2021-09-12

**Authors:** Yifang Ren, Huanhuan Lu, Longyan Chen, Simone Sabatelli, Chaojie Wang, Guanglin Xie, Ping Wang, Meike Liu, Wenkai Wang, Paolo Audisio

**Affiliations:** 1 Institute of Entomology, College of Agriculture, Yangtze University, Jingzhou 434025, China; 201973050@yangtzeu.edu.cn (Y.R.); clylyly@126.com (L.C.); 202071670@yangtzeu.edu.cn (C.W.); xieguanglin@yangtzeu.edu.cn (G.X.); wangping1992@yangtzeu.edu.cn (P.W.); 2 Chongqing Key Laboratory of Vector Insects, College of Life Sciences, Chongqing Normal University, Chongqing 401331, China; 2019110513046@stu.cqnu.edu.cn; 3 Dipartimento di Biologia e Biotecnologie “Charles Darwin”, Sapienza Università di Roma, Viale dell’Università 32, I-00185 Rome, Italy; simone.sabatelli@uniroma1.it (S.S.); paolo.audisio@uniroma1.it (P.A.)

**Keywords:** molecular, mitochondrial genome, genome structure, phylogenetic analysis

## Abstract

**Simple Summary:**

Many species of Cerambycidae are important pests in the agriculture, forestry, and fruit industries, and which have research significance. The subfamily Lamiinae is the most taxonomically diverse subfamily of Cerambycidae, but relationships between the tribes of Lamiinae are still unresolved. In order to provide a new perspective on the phylogenetic relationships among the tribes of Lamiinae, the mitogenomes of two species representing two tribes, *Agapanthia amurensis* (Agapanthiini) and *Moechotypa diphysis* (Ceroplesini), were sequenced. We present annotated, complete mitogenomes of these two species, and the results of a comparative analysis of both mitogenomes. The two new mitogenomes were found to be highly conservative, as found in other Cerambycidae. We also reconstructed the phylogenetic trees using mitogenomes of 38 species/subspecies of Lamiinae. Overall, this study explores the phylogenetic position between some tribes based on mitogenomic data and provides a further basis for studying the evolution of Lamiinae.

**Abstract:**

The subfamily Lamiinae is the most taxonomically diverse subfamily of Cerambycidae, but relationships between tribes of Lamiinae are still unresolved. In order to study the characteristics of the mitogenomes of Lamiinae and the tribal-level phylogenetic relationships, we sequenced the mitogenomes of two species representing two tribes, *Agapanthia amurensis* (Agapanthiini) and *Moechotypa diphysis* (Ceroplesini), with a total length of 15,512 bp and 15,493 bp, respectively. The gene arrangements of these two new mitogenomes were consistent with the inferred ancestral insect mitogenomes. Each species contained 37 typical mitochondrial genes and a control region (A + T-rich region), including 13 protein-coding genes (*PCGs*), 22 transfer RNA genes (tRNAs), and two ribosomal RNA genes (rRNAs). All *PCGs* initiated with the standard start codon ATN, and terminated with the complete stop codons of TAA and TAG, or incomplete stop codon T. All tRNAs could be folded into a clover-leaf secondary structure except for *trnS1*, in which the dihydrouridine (DHU) arm was reduced. Moreover, we studied the phylogenetic relationships between some tribes of Lamiinae based in mitochondrial *PCGs* in nucleotides; our results show that the relationships were as follows: (Onciderini + ((Apomecynini + Acanthocinini) + ((Ceroplesini + Agapanthiini) + ((Mesosini + Pteropliini) + ((Dorcaschematini + (Saperdini 1 + (Phytoeciini + Saperdini 2))) + (Batocerini + Lamiini)))))).

## 1. Introduction

The family Cerambycidae is one of the largest families of the superfamily Chrysomeloidea (Coleoptera: Polyphaga), consisting of a little less than 40,000 species, among which Lamiinae have about 20,000 species [1]. Thus, some species of Cerambycidae are important pests in the agriculture, forestry, and fruit industries. Some are also important quarantine pests. For example, some *Monochamus* species are the main transmission vectors of the pine wood nematode *Bursaphelenchus xylophilus* (Aphelenchoidae) [2,3]. However, adults of many species of Lepturinae in the Cerambycidae family have flower-visiting behaviors and help plants to pollinate [4]. All in all, Cerambycidae is believed to have great potential for applications in forest health and ecological biodiversity assessment [5,6,7].

As the most taxonomically diverse subfamily in Cerambycidae, the monophyly of Lamiinae is supported based on morphology and/or molecule studies [8,9,10,11,12,13]. However, the phylogenetic relationships of tribes among Lamiinae are controversial. Latreille (1825) proposed a modern classification of Cerambycidae, first using the term Lamiaires, which later became Lamiinae [14]. Blanchard (1845) proposed names for suprageneric groups of Lamiinae and recognized seven groups (Acanthocinites, Lamiites, Mesosites, Petrognathites, Saperdites, Stellognathites and Tetraophtalmites) [15]. Then, Thomson (1860) proposed an eight-rank taxonomic system (in French: Famille, Tribu, Sous-Tribu, Groupe, Sous-groupe, Division, Genre, Espèce), and recorded 17 groups of Lamiinae, which later increased to 33 groups in 1864 [16,17]. This laid a foundation for the later taxonomic study of tribes among Lamiinae. Aurivillius (1922, 1923) compiled a catalogue of Lamiinae and divided it into 96 tribes, which was the first comprehensive summary of Lamiinae [18,19]. Breuning (1958–1969) significantly reduced the tribes to 58 by synonymizing some controversial tribes [20,21,22,23,24,25,26,27,28,29,30,31], but this system was not fully accepted by subsequent researchers. More recently, Bouchard et al. (2011) synthesized the data of all known extant and fossil Coleoptera for the first time, and recognized 80 tribes of Lamiinae [32]. Souza et al. (2020) performed a study on the tribal classification of Lamiinae by molecular phylogenetic assessment [13]. They confirmed the monophyly of Lamiinae, and suggested some synonyms for its tribes based on the fragments of two mitochondrial genes (cytochrome c oxidase subunit 1 and large ribosomal RNA subunit) and three nuclear genes (wingless, carbamoyl-phosphate synthase domain of the *CAD* locus, and large ribosomal rRNA subunit).

Insect mitogenomes present unique features, such as maternal inheritance, low molecular weight, low recombination level, and fast evolutionary rate. Thus, they are widely used as molecular markers in studies of classification, genetic evolution, and phylogenetic analysis [33,34,35]. Insect mitogenomes consist of 37 genes, including 13 protein-coding genes (*PCGs*), 22 transfer RNA genes (tRNAs), two ribosomal RNA genes (rRNAs), and a control region (A + T-rich region) [33]. With the rapid development of next-generation sequencing, about 40,000 mitogenomes of animals have been published in the NCBI datasets [36]. However, few studies have been proposed based on molecular methods at the tribal level of Lamiinae. In this study, mitogenomes of two species of Lamiinae representing two tribes, *Agapanthia amurensis* Kraatz, 1879 (Agapanthiini) and *Moechotypa diphysis* (Pascoe, 1871) (Ceroplesini), were sequenced and analyzed. Of these, *M. diphysis* was the first complete mitogenome of Ceroplesini. In addition, we used the available and annotated mitogenomes from NCBI and two newly sequenced mitogenomes to infer the phylogenetic relationships between some tribes among Lamiinae.

## 2. Materials and Methods

### 2.1. Sample Preparation and DNA Extraction

Specimens of *Agapanthia amurensis* Kraatz, 1879 and *Moechotypa diphysis* (Pascoe, 1871) were collected from Enshi Tujia and Miao Autonomous Prefecture, Hubei Province, China (May 2020). The latitude and longitude of the collection sites are 30°36′18.6″ N and 110°4′6.8″ E. All specimens were identified by Prof. Guanglin Xie based on morphological characteristics. All specimens were immediately preserved in 100% ethanol and stored at −20 °C in the Entomological Museum of Yangtze University (No. *A*. *amurensis* YZU20200507134 and No. *M*. *diphysis* YZU20200507216). Then, total genomic DNA was extracted from the thoracic muscle tissues using a DNeasy DNA Blood & Tissue Kit (Qiagen, Beijing, China).

### 2.2. Sequence Analysis

Two mitogenome sequences were generated using the Illumina HiSeq platform with paired ends of 2 × 251 bp at Biomarker Technologies Co. Ltd. (Beijing, China). Raw reads were filtered, and quality was assessed using Fast-QC (http://www.bioinformatics.babraham.ac.uk/projects/fastqc, accessed on 01 July 2021) based on Q20 (>95%) and Q30 (>90%). After quality trimming, the reads were assembled by Geneious v8.1.3 (Biomatters, Auckland, New Zealand) [37] with default parameters and using the mitogenome of *Aromia bungii* (Faldermann, 1835) (GenBank accession no. MT371041) as reference [38]. All *PCGs* of two mitogenomes were identified based on finding the open reading frames (ORFs) and translated by Geneious v8.1.3 according to the invertebrate mitochondrial genetic code. The secondary structures of the tRNAs were identified by the MITOS Web Server [39] and drawn in Adobe Illustrator CC2019. The positions of rRNAs and control region were predicted by adjacent genes and homology alignment with *A*. *bungii*. The mitogenome maps were drawn using CGView Server [40]. Nucleotide composition, AT or GC skews, and relative synonymous codon usage (RSCU) were analyzed by PhyloSuite v1.2.2 [41]. The tandem repeats of the control region were predicted by the Tandem Repeats Finder online server (http://tandem.bu.edu/trf/trf.basic.submit.html, accessed on 01 July 2021) [42].

### 2.3. Phylogenetic Analysis

Two newly sequenced mitogenomes and 36 species/subspecies of Lamiinae were selected from the NCBI, representing 12 tribes (Acanthocinini, Agapanthiini, Apomecynini, Batocerini, Ceroplesini, Dorcaschematini, Lamiini, Mesosini, Onciderini, Phytoeciini, Pteropliini, Saperdini). In addition, two species (*Chrysomela vigintipunctata* and *Plagiodera versicolora*) of Chrysomelidae were selected as outgroups (Supplementary Materials Table S1). The *13PCGs* were aligned by MAFFT v7.0 [43]. Then, poorly aligned positions and high divergence regions were removed by Gblocks v0.91b in PhyloSuite. The potential index of substitution saturation (*Iss*) of each codon position of each nucleic acid sequence was analyzed by DAMBE v7.2.1 based on default parameters. [44]. Phylogenetic analyses were conducted using two datasets: *13PCGs* (nucleotides sequences for protein-coding genes; including all codon positions) and *13PCGs_AA* (amino acids of *13PCGs*). The phylogenetic analyses were reconstructed by the Bayesian inference (BI) and the Maximum likelihood (ML) methods based on these two datasets. The best evolutionary model of BI was inferred by PartitionFinder v2.1.1 [45] using the greedy search algorithm with branch lengths linked and Bayesian information criterion (BIC) in PhyloSuite. The best fit model of ML was selected using ModelFind in IQ-TREE. ML analysis was conducted by IQ-TREE [46], and node supports were computed via 1000 ultrafast bootstrap replicates. BI analysis was conducted by MrBayes v3.2.6 [47] under four Markov chain Monte Carlo (MCMC) chains of 1 million generations twice, sampled every 1000 generations, with the first 25% of generations removed as burn-in. MCMC analysis was stopped when the average standard deviation of the split frequency was below 0.01. Additionally, PhyloBayes analysis based on the CAT-GTR model was conducted by PhyloBayes v1.5a on CIPRES [12,48]. Two chains were run until the likelihood had satisfactorily converged (maxdiff < 0.2, minimum effective size > 50). The phylogenetic tree was displayed and edited by Tree of Life (iTOL, http://itol.embl.de, accessed on 01 July 2021) [49].

## 3. Results and Discussion

### 3.1. General Features of the Mitogenomes of Agapanthia amurensis and Moechotypa diphysis

**Figure 1:**
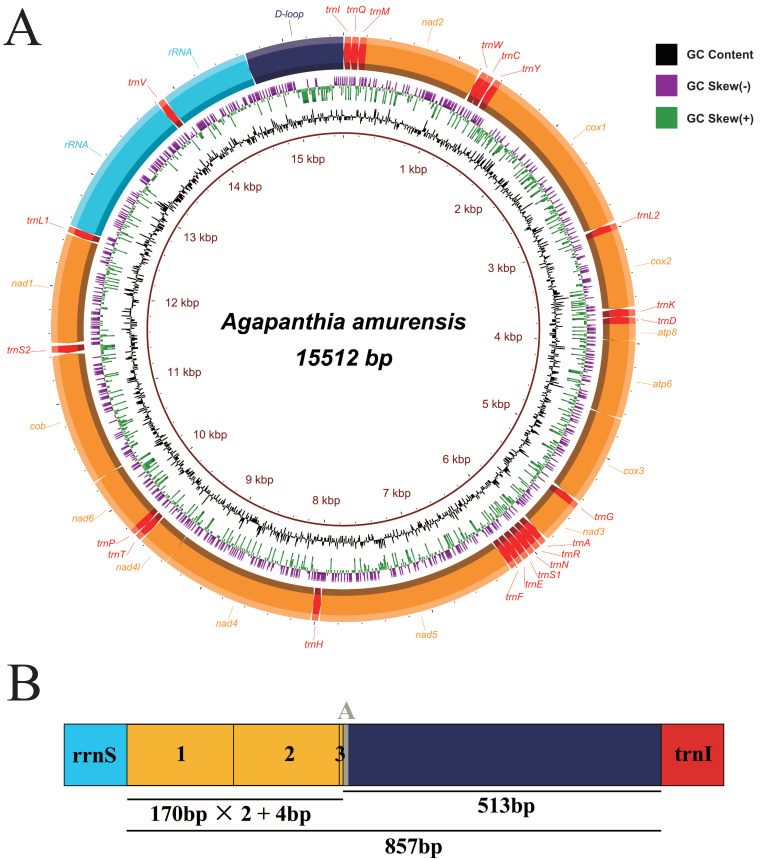
Complete mitogenome of *Agapanthia amurensis*. (**A**) Circular map. (**B**) Organization of the A + T-rich regions.

**Figure 2:**
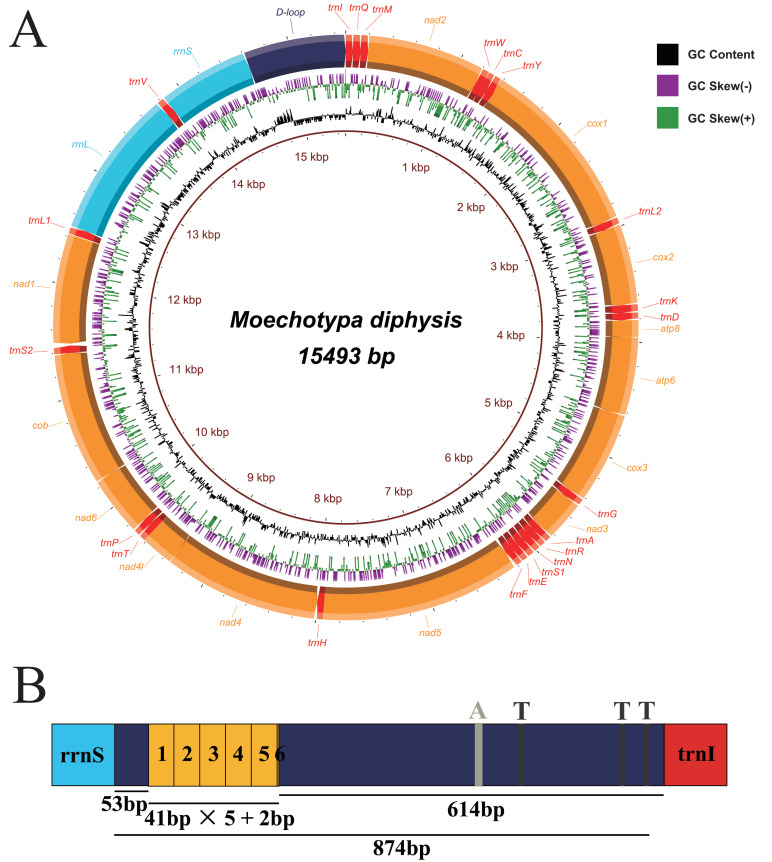
Complete mitogenome of *Moechotypa diphysis*. (**A**) Circular map. (**B**) Organization of the A + T-rich regions.

The complete mitogenomes of *Agapanthia amurensis* (GenBank accession number: MW617354) and *Moechotypa diphysis* (MW617356) were 15,512 bp and 15,493 bp in length, respectively (Table S1). Both mitogenomes included 37 typical mitogenomic genes and a control region. In these two mitogenomes, most genes (nine *PCGs* and 14 tRNAs) were concentrated on the J strand, and others (four *PCGs*, eight tRNAs, and two rRNAs) on the N strand (Figure 1a and Figure 2a, Table S2). Aside from the control region, six intergenic regions were found in the mitogenomes of both *A*. *amurensis* (35 bp total) and *M*. *diphysis* (31 bp total), and the longest region was detected between *trnS2* and *nad1*. Sixteen overlapping regions were found in the mitogenomes of both *A*. *amurensis* (50 bp total) and *M*. *diphysis* (45 bp total) (Table S3). The base composition of *A*. *amurensis* was A (38.5%), T (36.7%), G (9.3%), and C (15.5%), and the base composition of *M*. *diphysis* was A (37.6%), T (38.0%), G (9.5%), and C (14.9%). Both sequences displayed a high AT nucleotide bias, with A + T% of the whole sequence of 75.2% in *A*. *amurensis* and 75.6% in *M*. *diphysis*; these were similar to other sequenced Lamiinae species (Table S4). The composition skew analysis showed that the two new mitogenomes presented a negative GC skew. For AT skew, the value for *A*. *amurensis* was 0.024 and for *M*. *diphysis* was −0.005 (Table S4). Circular maps of the mitogenomes of these two new sequences were visualized in Figure 1a and Figure 2a.

### 3.2. Genome Structure

#### 3.2.1. Protein-Coding Genes

The *PCGs* ranged from 156 bp (*atp8*) to 1714 bp (*nad5*) in *A*. *amurensis*, and 156 bp (*atp8*) to 1720 bp (*nad5*) in *M*. *diphysis* (Table S4). *A*. *amurensis* and *M*. *diphysis* exhibited similar start and stop codons. All *PCGs* started with the typical ATN codon, and most *PCGs* ended with TAA or TAG, while three *PCGs* (*cox1*, *cox2*, and *nad5*) in *A*. *amurensis* and four *PCGs* (*cox1*, *cox2*, *nad4*, and *nad5*) in *M*. *diphysis* ended with incomplete stop codon T. In all 38 species/subspecies of Lamiinae, the termination TAA occurred more frequently than TAG (Table S2).

**Figure 3:**
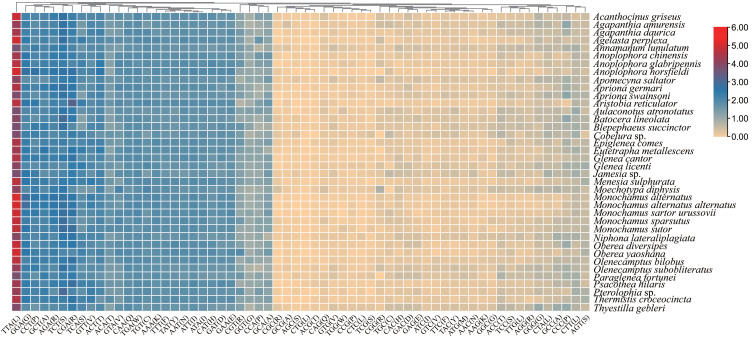
Relative synonymous codon usage (RSCU) of *PCGs* in mitogenomes of Lamiinae. X- and Y-axis represent codon type and species name, respectively.

Research on relative synonymous codon usage (RSCU) showed that the frequency of A or T is higher than that of G or C in the third codon position (Figure 3). For example, the third codon position of the seven most used codons (TTA, TCT, AGA, CCT, GTA, ACT, and TGT) in the mitogenome of *M*. *diphysis* was A or T, whereas codons with G or C in the third position (GCG, CCG, CGC, and GGC) were seldom presented in the mitogenome of *M*. *diphysis* (Table S5).

#### 3.2.2. Transfer and Ribosomal RNA Genes

**Figure 4:**
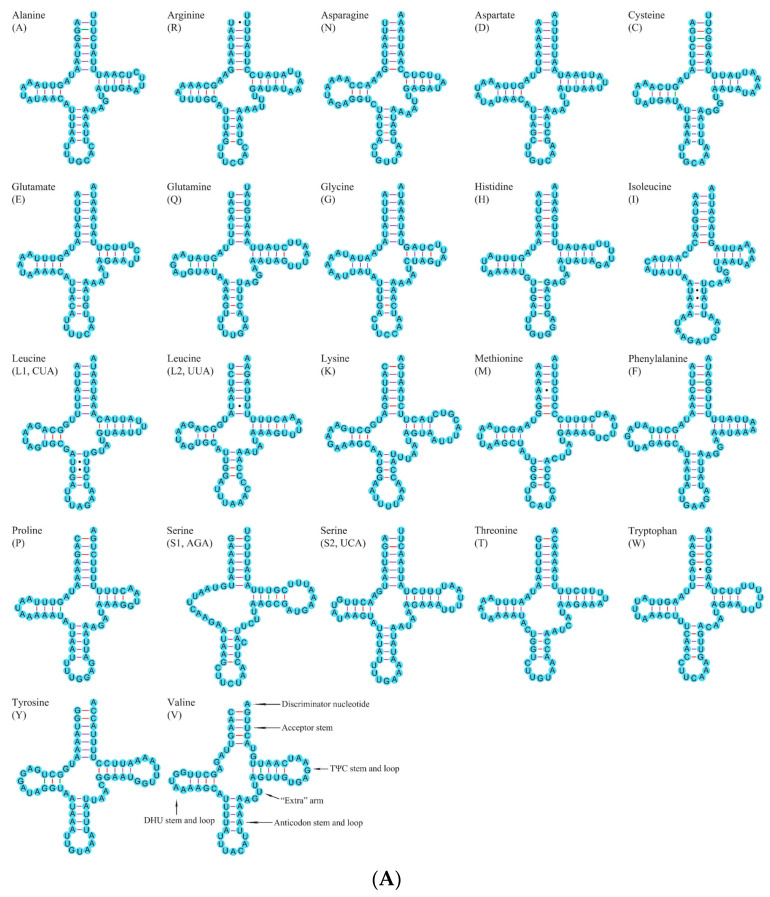

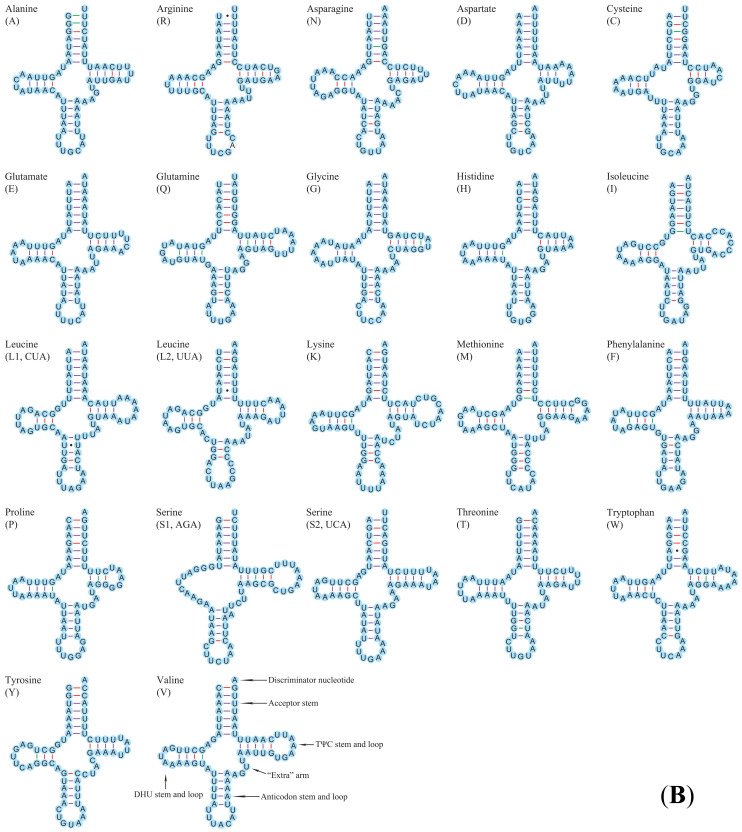
(**A**) Secondary structures of tRNAs in mitogenome of *Agapanthia amurensis*; bonds of A-U, G-C are marked with purple and red lines, and G-U mismatched bases with green lines and solid dots. (**B**) Secondary structures of tRNAs in mitogenome of *Moechotypa diphysis*; bonds of A-U, G-C are marked with purple and red lines, and G-U mismatched bases with green lines and solid dots.

Both new mitogenomes included 22 typical tRNAs genes. The size of these genes ranged from 64 bp (*trnE*, *trnG*, *trnL1*, and *trnP*) to 70 bp (*trnK*, and *trnY*) in *A. amurensis*, and from 61 bp (*trnC*) to 70 bp (*trnK*) in *M. diphysis* (Table S2). The whole tRNA region of these two mitogenomes was 1462 bp in *A. amurensis* and 1435 bp in *M. diphysis* (Table S4). Except for *trnS1*, which lacked a dihydrouracil (DHU) arm and formed a simple loop, other tRNAs could be folded into the classic clover-leaf secondary structure (Figure 4a,b). This phenomenon often occurs in the mitogenomes of Cerambycidae [50,51]. Based on the secondary structure of tRNAs, we found five types of mismatched bases (U-U, G-U, A-G, A-C, and A-A) in *A*. *amurensis*, and three types (U-U, G-U, and A-G) in *M*. *diphysis*.

There were two rRNAs in both new mitogenome sequences: *rrnL* (*16S*rRNA) was located between *trnL* and *trnV*, and *rrnS* (*12S*rRNA) was located between *trnV* and the control region. The length of *rrnL* was 1281 bp in *A*. *amurensis* and 1280 bp in *M*. *diphysis*, and the length of *rrnS* was 781 bp (*A*. *amurensis*) and 777 bp (*M*. *diphysis*), respectively (Table S2). These two rRNAs displayed a heavy AT nucleotide bias, with A + T content of 78.7% in *A*. *amurensis* and 80.4% in *M*. *diphysis*. Both rRNAs showed a negative AT skew and a positive GC skew in these two new sequences (Table S4).

#### 3.2.3. Control Region

The control region is normally the largest non-coding region in insect mitogenomes, also known as the A + T-rich region owing to the high AT content. This region was thought to have a function in regulating the transcription and replication of insect genes [52,53,54]. Tandem repeats in the control region were considered as the sequences that mainly affect the size of the mitogenome [52,55]. In this study, the control region of both new mitogenomes was located between *rrnS* and *trnI*, and the size was 857 bp in *A*. *amurensis* and 874 bp in *M*. *diphysis* (Table S2). One tandem repeat-like region was found in the control region of each. One Poly (A) was found in the non-repeat region of each. Three Poly (T) were found in non-repeat regions of *M*. *diphysis* (Figure 1b and Figure 2b); Poly (T) stretch was considered as an origin of transcription and replication [52].

### 3.3. Phylogenetic Analysis

**Figure 5:**
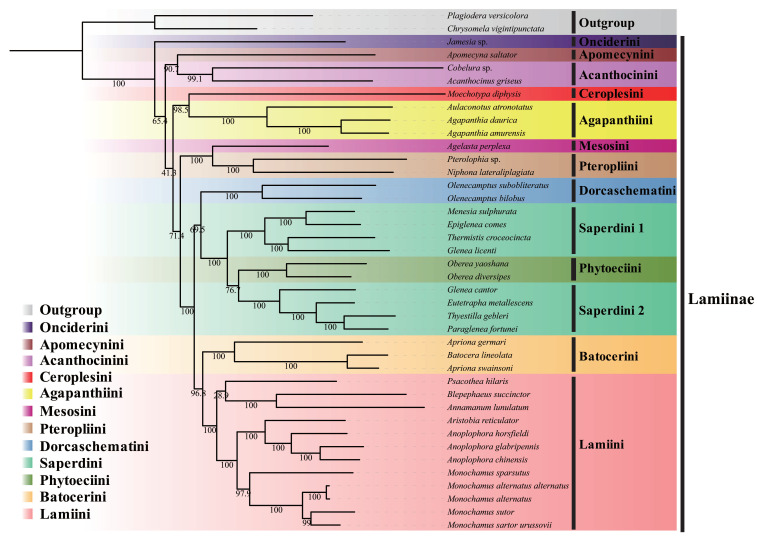

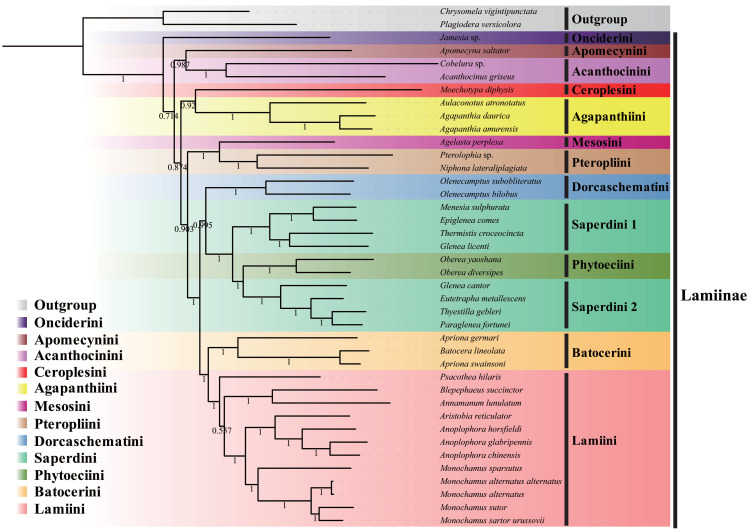
(**A**) Maximum likelihood (ML) phylogenetic tree based on *13PCGs* dataset. Numbers on branches are bootstrap values. (**B**) Bayesian inference (BI) phylogenetic tree based on *13PCGs* dataset. Numbers on branches are Bayesian posterior probabilities.

The substitution saturation analyses (Table S6) showed that the index of substitution saturation (*Iss*) was less than *Iss.cSym* (*p* < *0.05*) regardless of whether datasets were trimmed by Gblocks. This result suggested that all nucleotide positions of *PCGs* could provide useful information for the phylogenetic analysis. Based on the *13PCGs* and *13PCGs*_*AA* datasets, the phylogenetic relationships between the 12 tribes of Lamiinae were inferred from ML and BI analyses. The best model of the phylogenetic relationship is presented in Table S7. Inconsistent topologies were generated by ML and BI analyses based on the *13PCGs*_*AA* dataset (Supplementary Materials Figure S1). The positions of some tribes (Agapanthiini, Acanthocinini, Onciderini, Apomecynini, and Phytoeciini) in ML and BI trees were different, and there were some clade nodes had low support values (Figure S1). It may require more mitogenomes and other molecular evidence of Lamiinae to solve these problems [56,57]. Except for the position of *Psacothea hilaris*, the ML (Figure 5a) and Bl (Figure 5b) analyses based on the *13PCGs* dataset produced basically consistent topologies, and the support value of the BI tree was generally higher than that of the ML tree. They all indicated that the relationships among the 12 tribes of Lamiinae were: (Onciderini + ((Apomecynini + Acanthocinini) + ((Ceroplesini + Agapanthiini) + ((Mesosini + Pteropliini) + ((Dorcaschematini + (Saperdini 1 + (Phytoeciini + Saperdini 2))) + (Batocerini + Lamiini)))))). Due to the heterogeneity in base composition and evolutionary rates, phylogenetics research based on mitogenomes is still controversial [58,59,60]. Previous studies argued that using a CAT-GTR model in PhyloBayes was more suitable than other methods to reconstruct relationships at the subfamily level and above [12,59,60]. In this study, the PhyloBayes analysis based on the *13PCGs*_*AA* dataset (Figure S2) using the CAT-GTR model produced almost congruent topologies with the phylogenetic tree based on the *13PCGs*. The only differences were the position of clades Onciderini and (Mesosini + Pteropliini). Therefore, only the phylogenetic trees constructed using the *13PCGs* dataset are shown in this paper (Figure 5a,b).

In this study, the monophyly of Lamiinae was highly supported, which had been confirmed in previous studies [8,9,10,11,12,13,51,61]. We also studied the phylogenetic relationships between some tribes among Lamiinae, and the monophyly of Lamiini was supported (BS = 100; PP = 1). The boundary of Lamiini is controversial; some researchers have supported the retention of Lamiini and Monochamini [32,62,63], while others have proposed that the tribe Monochamini should be included in Lamiini [64,65,66]. Souza et al. (2020) proposed that Monochamini is a synonym of Lamiini based on the fragments of two mitochondrial genes (*cox1* and *rrnL*) and three nuclear genes (*Wg*, *CPS*, and *LSU*) [13], which is also supported by our present result. Moreover, in our study, the monophyly of the tribe Saperdini was not supported as Phytoeciini was nested within the Saperdini clade (BS = 76.7; PP = 1). Based on this result, we considered that Phytoeciini should be included in Saperdini. Souza et al. (2020), who performed the first, relatively dense phylogenetic systematic assessment of Lamiinae, supported Phytoeciini as a synonym of Saperdini [13]. Further, the phylogenetic tree (Figure 3 in Zhang et al. (2021)) of 30 species of Lamiinae based on the nucleotide dataset of the *13PCGs* of mitogenomes showed the same result [61].

## 4. Conclusions

This study describes two complete mitogenomes of *Agapanthia amurensis* and *Moechotypa diphysis* in Lamiinae. The gene arrangements of these new mitogenomes were consistent with other longhorn beetles. Each species contained 37 typical mitochondrial genes and a control region. All tRNAs could be folded into a clover-leaf secondary structure except for *trnS1*, in which the dihydrouridine (DHU) arm was reduced. In all 38 species/subspecies of Lamiinae in this study, the termination TAA occurred more than TAG. Our phylogenetic tree inferences of Lamiinae based on mitogenomes confirm the monophyly of Lamiinae. Regarding the relationships between the tribes among Lamiinae, our results support Lamiini being monophyletic and Phytoeciini as a synonym of Saperdini. In this study, the Bayesian analysis based on the *13PCGs* dataset is better than other analyses, which of course requires more samples and studies to verify. All in all, in order to better understand the phylogenetic relationships between tribes among Lamiinae, more mitochondrial datasets and other molecular evidence are still needed. Using more taxon samples and molecular markers may help to determine the higher-level relationships among Cerambycidae.

## Data Availability

All mitogenomic sequences in this study are available in the GenBank database (https://www.ncbi.nlm.nih.gov/nuccore, accessed on 01 July 2021), and the newly generated sequences are deposited in GenBank with accession numbers MW617354 and MW617356.

## Supplementary Materials

The following are available online at https://www.mdpi.com/article/10.3390/insects12090820/s1. Figure S1: Phylogenetic relationship of ML and Bl trees based on *13PCGs*_*AA* dataset. Figure S2: Phylogenetic relationship of PhyloBayes tree based on *13PCGs*_*AA* dataset. Table S1: Summary of mitogenome data used in this study. Table S2: Comparison of annotated mitogenomes of Lamiinae. Table S3: Intergenic region statistics of mitogenomes of Lamiinae. Table S4: Nucleotide composition of mitogenomes of *Agapanthia amurensis* and *Moechotypa diphysis*. Table S5: Relative synonymous codon usage (RSCU) of *PCGs* in mitogenomes. Table S6: Saturation substitution tests for *13PCGs* dataset. Table S7: Best model of phylogenetic relationships.
