# Supplementary material for: Comparative Mitogenomic Analysis of Two Longhorn Beetles (Coleoptera: Cerambycidae: Lamiinae) with Preliminary Investigation into Phylogenetic Relationships of Tribes of Lamiinae
Source: Insects. 2021 Sep 12;12(9):820. doi: 10.3390/insects12090820 (PMC8471637; doi:10.3390/insects12090820)

1. The phylogenetic Maximum Likelihood (ML) tree based on *13PCGs_AA* dataset.

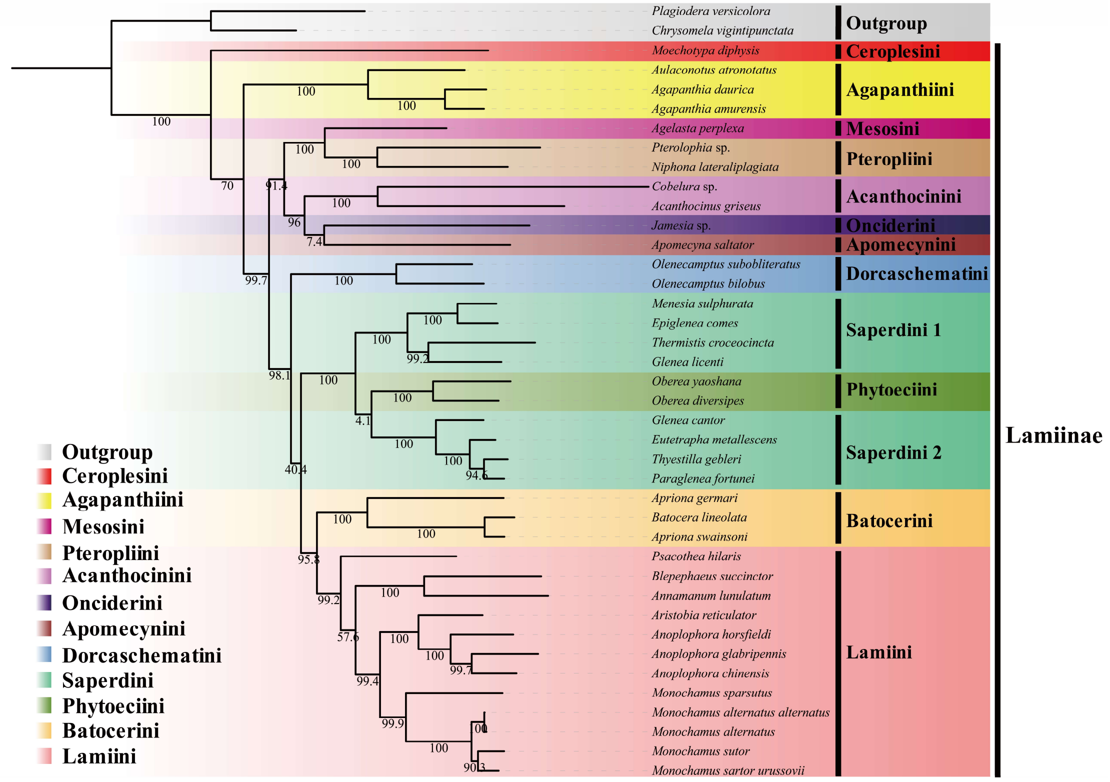

2. The phylogenetic Bayesian Inference (BI) tree based on *13PCGs_ AA* dataset.

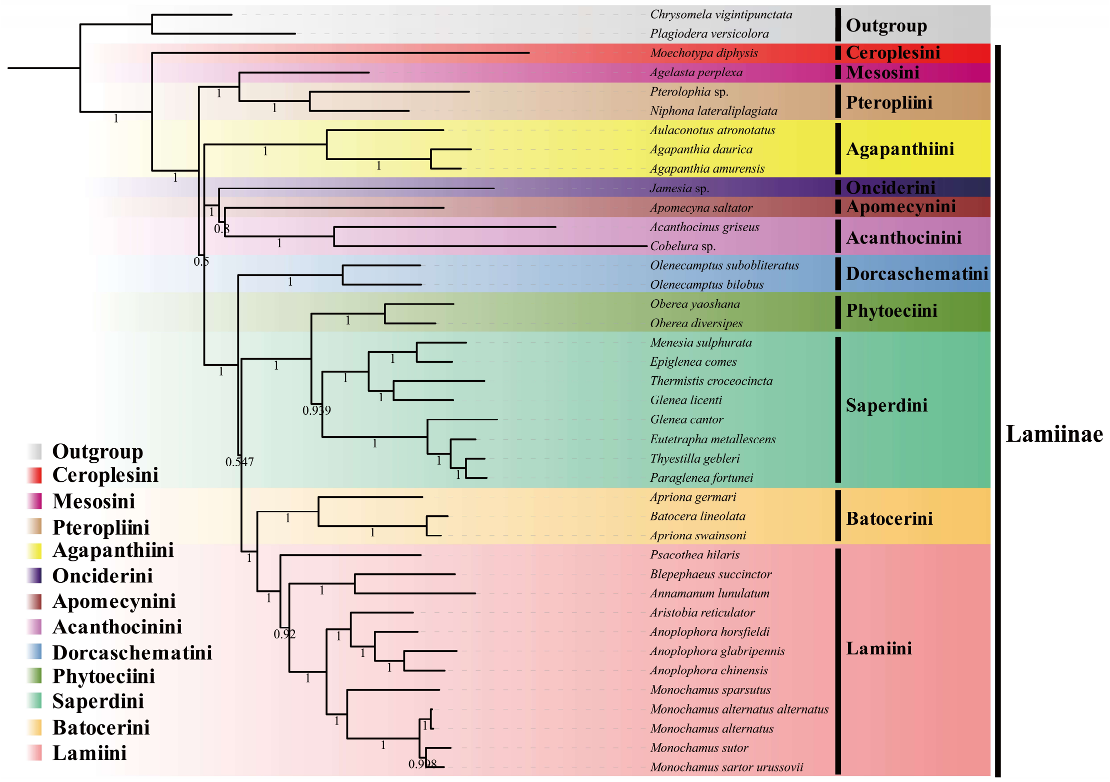

Supplement: Supplementary file 1 [file insects-12-00820-s001.zip › Figure S1 Phylogenetic relationship of the ML and Bl trees based on 13PCGs_AA dataset.pdf]
