# Supplementary figures and images for: Comparative Mitogenomic Analysis of Two Longhorn Beetles (Coleoptera: Cerambycidae: Lamiinae) with Preliminary Investigation into Phylogenetic Relationships of Tribes of Lamiinae
Source: Insects. 2021 Sep 12;12(9):820. doi: 10.3390/insects12090820 (PMC8471637; doi:10.3390/insects12090820)

1. The Phylogenetic relationship of PhyloBayes tree based on *13PCGs\_AA* dataset.

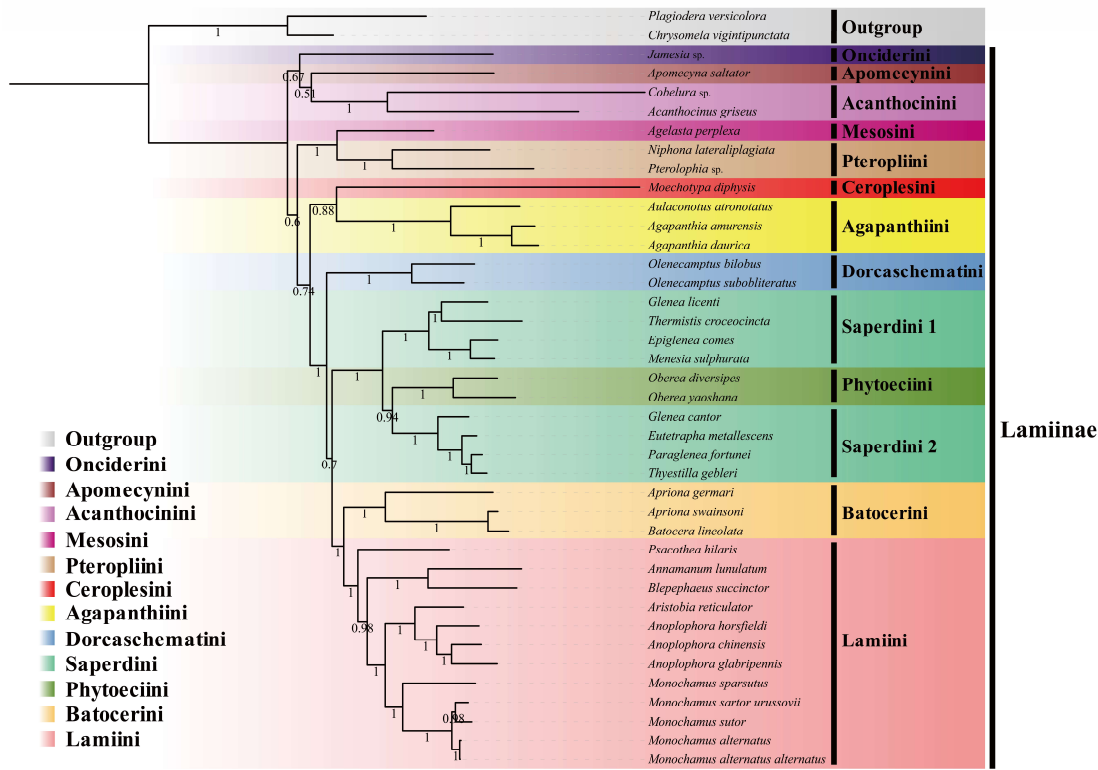

Supplement: Supplementary file 1 [file insects-12-00820-s001.zip › Figure S2 Phylogenetic relationship of PhyloBayes tree based on 13PCGs_AA dataset.pdf]
